# Factors associated with native heart survival and intermediate-term outcomes in acute myocardial infarction-related cardiogenic shock

**DOI:** 10.1093/ehjopen/oeag015

**Published:** 2026-02-26

**Authors:** Christos P Kyriakopoulos, Konstantinos Sideris, Ioannis Kyriakoulis, Iosif Taleb, Eleni Maneta, Chong Zhang, Eleni Tseliou, Spencer Carter, Roberta Florido, Frederick G Welt, Josef Stehlik, Craig H Selzman, James C Fang, Matthew L Goodwin, Joseph E Tonna, Thomas C Hanff, Stavros G Drakos

**Affiliations:** Division of Cardiovascular Medicine, Department of Internal Medicine, University of Utah Health & School of Medicine, 30 North Mario Capecchi Dr., Salt Lake City, UT 84112, USA; Nora Eccles Harrison Cardiovascular Research and Training Institute, University of Utah, 95 South 2000 East, Salt Lake City, UT 84132, USA; Division of Cardiovascular Medicine, Department of Internal Medicine, University of Utah Health & School of Medicine, 30 North Mario Capecchi Dr., Salt Lake City, UT 84112, USA; Nora Eccles Harrison Cardiovascular Research and Training Institute, University of Utah, 95 South 2000 East, Salt Lake City, UT 84132, USA; Division of Cardiovascular Medicine, Department of Internal Medicine, University of Utah Health & School of Medicine, 30 North Mario Capecchi Dr., Salt Lake City, UT 84112, USA; Nora Eccles Harrison Cardiovascular Research and Training Institute, University of Utah, 95 South 2000 East, Salt Lake City, UT 84132, USA; Division of Cardiovascular Medicine, Department of Internal Medicine, University of Utah Health & School of Medicine, 30 North Mario Capecchi Dr., Salt Lake City, UT 84112, USA; Nora Eccles Harrison Cardiovascular Research and Training Institute, University of Utah, 95 South 2000 East, Salt Lake City, UT 84132, USA; Division of Cardiovascular Medicine, Department of Internal Medicine, University of Utah Health & School of Medicine, 30 North Mario Capecchi Dr., Salt Lake City, UT 84112, USA; Nora Eccles Harrison Cardiovascular Research and Training Institute, University of Utah, 95 South 2000 East, Salt Lake City, UT 84132, USA; Division of Epidemiology, Department of Internal Medicine, University of Utah Health & School of Medicine, 30 North Mario Capecchi Dr., Salt Lake City, UT 84112, USA; Division of Cardiovascular Medicine, Department of Internal Medicine, University of Utah Health & School of Medicine, 30 North Mario Capecchi Dr., Salt Lake City, UT 84112, USA; Nora Eccles Harrison Cardiovascular Research and Training Institute, University of Utah, 95 South 2000 East, Salt Lake City, UT 84132, USA; Division of Cardiovascular Medicine, Department of Internal Medicine, University of Utah Health & School of Medicine, 30 North Mario Capecchi Dr., Salt Lake City, UT 84112, USA; Division of Cardiovascular Medicine, Department of Internal Medicine, University of Utah Health & School of Medicine, 30 North Mario Capecchi Dr., Salt Lake City, UT 84112, USA; Division of Cardiovascular Medicine, Department of Internal Medicine, University of Utah Health & School of Medicine, 30 North Mario Capecchi Dr., Salt Lake City, UT 84112, USA; Division of Cardiovascular Medicine, Department of Internal Medicine, University of Utah Health & School of Medicine, 30 North Mario Capecchi Dr., Salt Lake City, UT 84112, USA; Nora Eccles Harrison Cardiovascular Research and Training Institute, University of Utah, 95 South 2000 East, Salt Lake City, UT 84132, USA; Division of Cardiothoracic Surgery, Department of Surgery, University of Utah Health & School of Medicine, 30 North Mario Capecchi Dr., Salt Lake City, UT 84112, USA; Division of Cardiovascular Medicine, Department of Internal Medicine, University of Utah Health & School of Medicine, 30 North Mario Capecchi Dr., Salt Lake City, UT 84112, USA; Division of Cardiothoracic Surgery, Department of Surgery, University of Utah Health & School of Medicine, 30 North Mario Capecchi Dr., Salt Lake City, UT 84112, USA; Division of Cardiothoracic Surgery, Department of Surgery, University of Utah Health & School of Medicine, 30 North Mario Capecchi Dr., Salt Lake City, UT 84112, USA; Division of Cardiovascular Medicine, Department of Internal Medicine, University of Utah Health & School of Medicine, 30 North Mario Capecchi Dr., Salt Lake City, UT 84112, USA; Division of Cardiovascular Medicine, Department of Internal Medicine, University of Utah Health & School of Medicine, 30 North Mario Capecchi Dr., Salt Lake City, UT 84112, USA; Nora Eccles Harrison Cardiovascular Research and Training Institute, University of Utah, 95 South 2000 East, Salt Lake City, UT 84132, USA

**Keywords:** Acute myocardial infarction, Cardiac assist device, Cardiogenic shock, Heart transplantation, Mechanical circulatory support, Survival

## Abstract

**Aims:**

Cardiogenic shock (CS) is the leading cause of in-hospital mortality in patients suffering acute myocardial infarction (AMI). Despite advances in their management, short- and long-term mortality remain unacceptably high. We assessed short and intermediate-term outcomes for a contemporary cohort of patients with AMI-CS managed at a referral centre with a large catchment area, and sought to identify clinical factors portending a favourable prognosis.

**Methods and results:**

Of 1162 consecutive, unselected patients with CS we studied 316 with AMI-CS. Our primary endpoint was native heart survival (NHS) defined as survival to discharge without advanced heart failure (HF) therapies. Our secondary endpoints were adverse events, overall survival, and readmissions up to 1 year following discharge. Association of clinical data with NHS was analysed using logistic regression. Of 316 patients, 168 (53.2%) achieved NHS, 140 (44.3%) died, and 8 (2.5%) were discharged after receiving advanced HF therapies. Overall, 181 patients (57.3%) received temporary mechanical circulatory support (MCS), with 78 (24.7%) receiving intra-aortic balloon pump, 107 (33.9%) percutaneous ventricular assist device, and 62 (19.6%) veno-arterial extracorporeal membrane oxygenation. Of 176 discharged patients (55.7%), 170 (53.8%) were alive at 30 days, and 156 (49.4%) at 1-year post-discharge, while 56 (31.8%) had at least one readmission and 30 (17.0%) one HF-related readmission, by 1-year post-discharge. Patients with NHS were younger, had lower CS severity by SCAI stage, less commonly underwent intubation, or received temporary MCS, had a shorter time from CS onset to MCS deployment, and more commonly underwent coronary intervention with fewer stents deployed, compared to patients who died or underwent advanced HF therapies. Bleeding and vascular complications were less common in patients achieving NHS compared to patients who died or received advanced HF therapies. After multivariable adjustments, clinical variables associated with NHS included: younger age, lower vasoactive-inotropic score, lower serum creatinine, and lactate at shock onset, successful coronary intervention with fewer stents deployed, and absence of intubation, or use of veno-arterial extracorporeal membrane oxygenation (all *P* ≤ 0.05).

**Conclusion:**

We studied a contemporary cohort of patients with AMI-CS and high rates of temporary MCS use, and identified clinical factors associated with a higher likelihood for successful outcomes. The need for transfer to an advanced CS centre, the impact and management of adverse events, and the type and timing of temporary MCS as opposed to intensification of pharmacologic therapy, should be studied as clinical practice targets for improving patient outcomes.

## Graphical Abstract

**Graphical Abstract:**
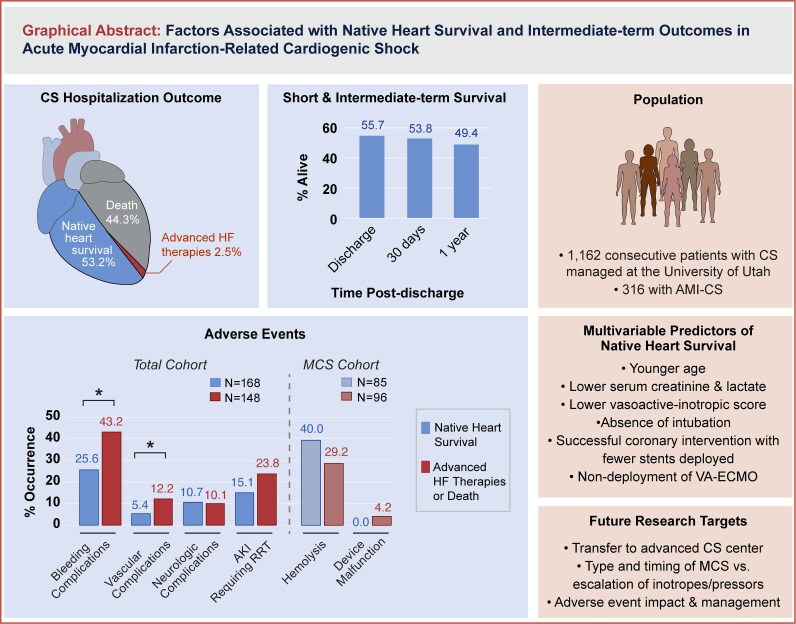

## Introduction

Cardiogenic shock (CS) is a clinical syndrome characterized by inadequate tissue perfusion and end-organ dysfunction stemming from severely impaired cardiac output. Based on the underlying aetiology, it is categorized into CS secondary to acute myocardial infarction (AMI-CS) or decompensated heart failure (HF).^1–5^ These two phenotypes have been shown to differ not only in terms of the underlying pathophysiology but also presentation and clinical course, response to treatment modalities, and outcomes.^4–8^

The SHOCK (Should We Emergently Revascularize Occluded Coronaries for Cardiogenic Shock) trial showcased a survival benefit with early revascularization in patients suffering AMI-CS.^9^ Since then and despite advances in patient management, outcomes remain poor with overall mortality approaching 40% at 30 days, and 50% at 1 year.^1,10–12^ Temporary mechanical circulatory support (MCS) devices have been increasingly used despite limited evidence of superiority. After multiple studies failing to confer a survival benefit with the use of intra-aortic ballon pump (IABP), percutaneous ventricular assist device (pVAD), or veno-arterial extracorporeal membrane oxygenation (VA-ECMO),^12–18^ the DanGer (Danish-German) Shock trial was the first to support the use of pVAD in AMI-CS,^19^ A recent study, however, suggested that only one-third of contemporary patients with AMI-CS in North America would fit the trial population characteristics.^20^ At the same time, high adverse event rates associated with the use of temporary MCS, have implications not only for outcomes of patients with AMI-CS but also for clinical management and systems of care.^21,22^

To assess outcomes of unselected patients and encompass clinical practices employed in different level of care institutions, we studied consecutive patients with AMI-CS who presented or were transferred to our referral centre serving a large catchment area, and captured clinical data pertaining to both outside and referral institution management. We assessed survival, major cardiac interventions, adverse events, and hospital readmission rates up to 1 year following index hospitalization discharge. We sought to identify clinical characteristics and management practices associated with a higher likelihood for a favourable outcome.

## Methods

### Study population

**Figure 1:**
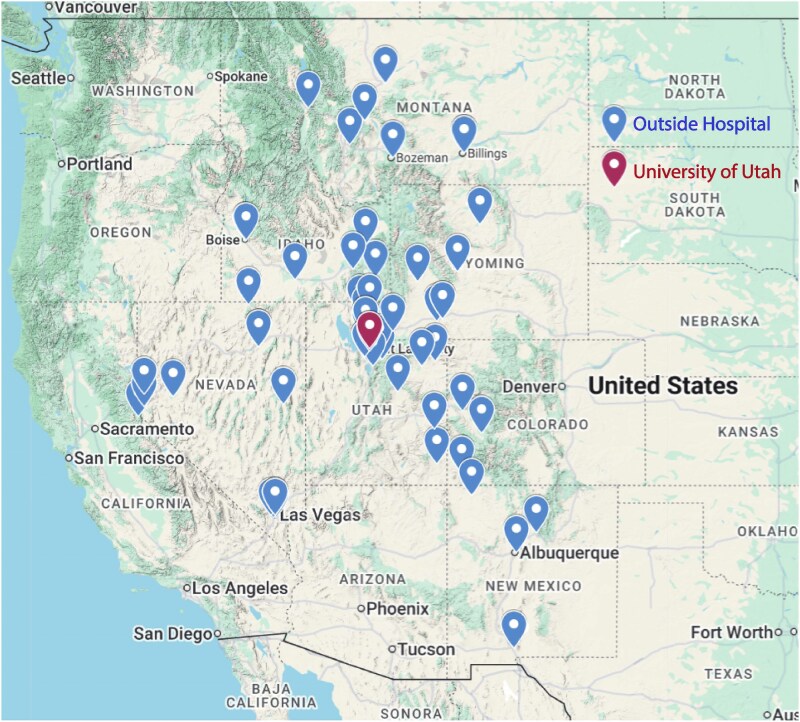
Outside healthcare facilities and University of Utah Hospital location map. Created using the Google Maps application interface.

We evaluated consecutive, prospectively enrolled, and retrospectively reviewed patients with CS, managed at the University of Utah Hospital from April 2015 to December 2021, and followed until December 2022. Our study cohort comprised patients with AMI-CS who presented or were transferred to our centre. *Figure 1* depicts the locations of the referring institutions and was created using the Google Maps application interface. The University of Utah Hospital is a quaternary healthcare institution providing 24-7 advanced cardiovascular care including dedicated cardiac intensive care management, primary percutaneous coronary intervention and coronary artery bypass graft surgery, temporary MCS with IABP, pVAD (i.e. Impella^®^; Abiomed, Danvers, MA, USA), temporary ventricular assist devices (VAD) (CentriMag™; Abbott Laboratories, Abbott Park, IL, USA), percutaneous ECMO including veno-arterial (VA-ECMO) and veno-pulmonary (TandemHeart^®^ and ProtekDuo^®^; LivaNova PLC, London, United Kingdom), and advanced HF therapies including durable left VAD (LVAD) implantation and heart transplantation (HTx). The Utah Cardiac Recovery Shock Team was established in April 2015 to evaluate patients suffering CS with a standardized comprehensive multidisciplinary assessment, as described previously.^23^ Patients with post-cardiotomy CS or multifactorial shock aetiologies (hypovolemic, vasodilatory, or septic shock preceding CS or sepsis developing within 48 h of CS onset) were excluded. Sepsis was defined by a positive blood, urine, or sputum culture or new antibiotic therapy due to high clinical suspicion despite negative cultures.

### Data collection and definitions

The study was approved by the University of Utah Institutional Review Board (IRB). Informed consent was obtained under IRB 80080 (Utah Cardiac Recovery Shock Registry), or waiver of consent was granted under IRB 72747 (Clinical Analyses in Cardiovascular Medicine). Clinical data were collected via chart review of electronic medical records of the University of Utah Hospital and outside institutions, and included patient demographics, baseline laboratory and haemodynamic data recorded closest to and within 8 h of shock onset, and imaging data collected closest to and within 24 h of shock onset. As shock onset, was defined the first time the patient met the CS definition below.

CS was defined using clinical and haemodynamic criteria as previously described,^9,24^ including one of the following: (i) systolic blood pressure (SBP) < 90 mmHg for >30 min, (ii) need for inotropes/vasopressors to maintain an SBP >90 mmHg, plus one of the following: (i) pulmonary capillary wedge pressure or left ventricular end diastolic pressure >15 mmHg and cardiac index <2.2 L/min/m^2^, (ii) clinical or radiological signs of pulmonary oedema, and (iii) impaired end-organ perfusion defined as altered mental status, cold clammy skin and extremities, or oliguria with urine output <30 mL/h.

Based on the underlying CS pathophysiology patients were stratified into AMI-CS or HF-related CS.^24^ AMI-CS included ST-segment elevation and non-ST-segment elevation MI, while HF-related CS included ischaemic or various types of non-ischaemic cardiomyopathy. Both patients with AMI or HF-related CS were further classified based on the chronicity of disease into acute or acute-on-chronic, with the latter having a prior diagnosis of HF. CS severity by Society for Cardiovascular Angiography and Interventions (SCAI) stages was assessed by the updated Cardiogenic Shock Working Group (CSWG) criteria,^25^ which has been previously validated in our center.^26^ The vasoactive-inotropic score (VIS) was calculated based on number and dosage of inotropes and vasopressors at shock onset.^27^

We used the Society of Thoracic Surgeons Interagency Registry for Mechanically Assisted Circulatory Support definitions^28^ for capturing the following adverse events: (i) neurologic complications including ischaemic stroke, haemorrhagic stroke, or transient ischaemic attack, (ii) bleeding complications including type 3 (overt bleeding accompanied by haemoglobin drop of 3 to <5 g/dL, or provided haemoglobin drop is related to bleed, or any transfusion with overt bleeding) or type 4 (infusion of ≥4 units of packed red blood cells within any 48 h during the first 7 days post-MCS implant), (iii) haemolysis defined as plasma-free haemoglobin values >20 mg/dL or serum lactate dehydrogenase levels >2.5 times the upper limit of normal, occurring >72 h post-MCS implant, and (iv) MCS device malfunction, defined as failure of one or more of the components of the MCS system which either directly causes or could potentially induce a state of inadequate circulatory support or death. We also captured acute kidney injury (AKI) requiring renal replacement therapy (RRT) and the following vascular access site complications: surgical or transcatheter vascular repair, fasciotomy for acute compartment syndrome, or amputation. We compared complication rates between groups in the full cohort and separately in the cohort of patients who underwent temporary MCS due to potential contribution to bleeding, vascular, neurologic complications, and AKI requiring RRT.^13,21,29–31^

### Study endpoints

Our primary endpoint was native heart survival (NHS) defined as survival to hospital discharge without receiving advanced HF therapies during index hospitalization (i.e. LVAD implantation or HTx).^6,7,32,33^ Our secondary outcomes were adverse events, overall survival (death from all causes), overall survival stratified by SCAI stage and MI location (left anterior descending artery vs. other coronary artery), major cardiac interventions, and hospital readmissions within 1 year following index hospitalization discharge.

### Statistical analysis

Demographics and clinical data were descriptively summarized and stratified by the outcome. Continuous variables were summarized as mean and standard deviation (SD) or median and interquartile range (IQR). Categorical variables were summarized as frequency and percentage. We compared categorical variables using the chi-squared or Fisher’s exact test and continuous variables using the *t*-test, Wilcoxon rank sum test, analysis of variance, or Kruskal–Wallis test, between patients achieving NHS and patients who either died or survived after undergoing advanced HF therapies.

Association with the primary outcome (NHS) was analysed using logistic regression. Explanatory variables were selected based on prior medical literature^6,8,34–40^ and significance in the unadjusted comparisons. Two models were constructed. The first model included variables collected at baseline (shock onset), while the second model included clinical parameters representing overall stay and management. Due to sample size limitations, we removed variables correlated with other variables in the model, had large amount of missingness, and lack of significance when adjusting for other variables in the model. Multicollinearity among variables was assessed using the variance inflation factor, with variables exhibiting multicollinearity being removed from the models. Missing values were imputed using multiple imputation with chained equations.^41^ Model coefficients and their standard errors were pooled across 10 multiply imputed datasets. Odds ratios (OR) (both univariable and multivariable) were reported with 95% confidence intervals (CI). Significance was determined at the *P* = 0.05 level and all tests were two sided. A Kaplan–Meier survival curve up to 1 year following index hospitalization discharge was generated. Statistical analyses were conducted in R 4.2.1.^42^

## Results

After excluding patients with multifactorial shock aetiologies or post-cardiotomy CS, 1162 patients were evaluated. Our study cohort comprised 316 patients suffering AMI-CS with 181 (57.3%) undergoing temporary MCS. Seventy-eight (24.7%) patients underwent support with an IABP, 107 (33.9%) with a pVAD, and 62 (19.6%) with VA-ECMO. Overall, 168 (53.2%) patients were discharged with a native heart, 140 (44.3%) died during index hospitalization, and eight (2.5%) were discharged after receiving advanced HF therapies. During index hospitalization, nine patients (6.1%) underwent durable LVAD implantation with one not surviving to discharge, while none underwent HTx.

Demographics and baseline clinical characteristics in patients discharged with a native heart vs. patients who died or received advanced HF therapies are presented in *Table 1* and Supplementary material online, *Table S1*. Patients with NHS were younger (62 ± 12 vs. 66 ± 13 years, *P* = 0.002), had lower CS severity at shock onset as evidenced by SCAI-CSWG stage (stage E: 39.9 vs. 53.4%, overall *P* = 0.035), and numerically lower rates of cardiac arrest (31.0 vs. 41.2%, *P* = 0.06), compared to patients who died during index hospitalization or survived after receiving advanced HF therapies. Patients with NHS less commonly underwent intubation (62.5 vs. 89.2%, *P* < 0.001), received temporary MCS overall (50.6 vs. 64.9%, *P* = 0.011), pVAD (26.8 vs. 41.9%, *P* = 0.005), VA-ECMO (11.3 vs. 29.1%, *P* < 0.001), or required escalation from IABP (18.2 vs. 47.1%, *P* = 0.006), compared to patients who died or underwent advanced HF therapies. Patients who achieved NHS compared to patients who died or underwent advanced HF therapies had a shorter time interval from CS onset to MCS deployment [90 (30, 270) vs. 152 (60, 511) min, *P* = 0.009] with the rates of MCS deployment at an outside hospital being comparable (47.1 vs. 36.5%, *P* = 0.15).

**Table 1:** Baseline clinical characteristics in patients stratified by in-hospital outcome

Variable	Native heart survival (N = 168)	Survival post-advanced HF therapies or death (N = 148)	*P*-value
**Demographics**			
**Age (years)—**Mean (SD)	62 (12)	66 (13)	0.002^t^
**Sex—**Female	44 (26.2%)	40 (27.0%)	0.87^c^
**Race—**White	140 (87.0%)	120 (87.0%)	0.36^f^
Black/African American	1 (0.6%)	1 (0.7%)	—
American Indian/Alaska Native	4 (2.5%)	3 (2.2%)	—
Native Hawaiian/Other Pacific Islander	0 (0.0%)	4 (2.9%)	—
Asian	3 (1.9%)	2 (1.4%)	—
Other	13 (8.1%)	8 (5.8%)	—
**Ethnicity—**Hispanic/Latino	13 (8.0%)	8 (6.4%)	0.60^c^
**Body mass index (kg/m^2^)—**Median (IQR)	29 (26, 32)	29 (26, 33)	0.86^w^
**Cardiogenic shock management and treatment**			
**Transfer from outside hospital**	122 (72.6%)	104 (70.3%)	0.64^c^
**Shock onset hospital—**University of Utah Hospital	100 (59.5%)	88 (59.5%)	0.99^c^
Outside hospital	68 (40.5%)	60 (40.5%)	—
**University of Utah hospital length of stay (days)—**Median (IQR)	10 (7, 20)	4 (1, 14)	<0.001^w^
**Outside hospital length of stay (days)—**Median (IQR)	1 (0, 2)	1 (0, 2)	0.83^w^
**Chronicity of disease—**Acute heart failure	149 (88.7%)	134 (90.5%)	0.59^c^
Acute-on-chronic heart failure	19 (11.3%)	14 (9.5%)	—
**Acute coronary syndrome type—**NSTEMI	49 (29.2%)	51 (34.5%)	0.31^c^
STEMI	119 (70.8%)	97 (65.5%)	—
**SCAI-CSWG stage at shock onset—**B	14 (8.3%)	9 (6.1%)	0.035^c^
C	53 (31.5%)	28 (18.9%)	—
D	34 (20.2%)	32 (21.6%)	—
E	67 (39.9%)	79 (53.4%)	—
**Cardiac arrest**	52 (31.0%)	61 (41.2%)	0.06^c^
**Inotropes/vasopressors at shock onset**	104 (61.9%)	91 (61.5%)	0.94^c^
**Vasoactive inotropic score—**Median (IQR)	4.2 (0.0, 14.3)	9.1 (0.0, 21.8)	0.08^w^
**Intubation**	105 (62.5%)	132 (89.2%)	<0.001^c^
**Temporary MCS**	85 (50.6%)	96 (64.9%)	0.011^c^
**Time to first temporary MCS (min)—**Median (IQR)**^a^**	90 (30, 270)	152 (60, 511)	0.009^w^
**Temporary MCS implant at outside hospital^a^**	40 (47.1%)	35 (36.5%)	0.15^c^
**IABP**	44 (26.2%)	34 (23.0%)	0.51^c^
**Escalation from IABP^a^**	8 (18.2%)	16 (47.1%)	0.006^c^
**Impella**	45 (26.8%)	62 (41.9%)	0.005^c^
**Escalation from Impella^a^**	10 (22.2%)	14 (22.6%)	0.97^c^
**VA-ECMO**	19 (11.3%)	43 (29.1%)	<0.001^c^
**Heart transplantation**	0 (0%)	0 (0%)	1.00^f^
**Durable LVAD implantation**	0 (0%)	9 (6.1%)	<0.001^f^
**Past medical history**			
**Hypertension**	108 (64.3%)	100 (69.9%)	0.29^c^
**Diabetes mellitus**	55 (32.7%)	56 (39.2%)	0.24^c^
**Smoking—**Current smoker	43 (25.7%)	24 (16.9%)	0.09^c^
Former smoker	44 (26.3%)	34 (23.9%)	—
Non-smoker	80 (47.9%)	84 (59.2%)	—
**Hyperlipidaemia**	77 (45.8%)	75 (52.4%)	0.24^c^
**Chronic kidney disease stage III–V**	13 (7.7%)	18 (12.6%)	0.15^c^
**Coronary artery disease**	60 (35.7%)	57 (39.9%)	0.45^c^
**Prior myocardial infarction**	35 (20.8%)	37 (25.9%)	0.29^c^
**Chronic heart failure**	19 (11.3%)	14 (9.5%)	0.59^c^
**Reduced LV ejection fraction (<50%)^a^**	17 (89.5%)	13 (92.9%)	1.00^f^
**Haemodynamics at Shock Onset**			
**Mean arterial blood pressure (mmHg)—**Median (IQR)	84 (75, 97)	82 (73, 94)	0.52^w^
**Mean right atrial pressure (mmHg)—**Median (IQR)	13 (9, 16)	13 (11, 19)	0.08^w^
**Pulmonary artery mean pressure (mmHg)—**Median (IQR)	30 (25, 39)	34 (25, 41)	0.17^w^
**Pulmonary capillary wedge pressure (mmHg)—**Median (IQR)	21 (17, 27)	24 (18, 30)	0.06^w^
**Cardiac index by fick (L/min/m^2^)—**Median (IQR)	1.9 (1.6, 2.5)	2.0 (1.5, 2.4)	0.84^w^
**Cardiac index by thermodilution (L/min/m^2^)-**Median (IQR)	1.9 (1.5, 2.6)	1.8 (1.2, 2.0)	0.016^w^
Systemic vascular resistance (dynes*s/cm^5^)—Median (IQR)	1284 (873, 1659)	1327 (848, 1609)	0.78^w^
Pulmonary vascular resistance (dynes*s/cm^5^)—Median (IQR)	182 (102, 257)	157 (105, 334)	0.92^w^
**Laboratory assessment at shock onset**			
**Serum creatinine (mg/dL)—**Median (IQR)	1.2 (1.0, 1.6)	1.5 (1.1, 2.0)	<0.001^w^
**Alanine transaminase (mg/dL)—**Median (IQR)	52 (29, 121)	68 (30, 207)	0.13^w^
**Total bilirubin (mg/dL)—**Median (IQR)	0.8 (0.5, 1.1)	0.7 (0.5, 1.1)	0.41^w^
**Blood glucose (mg/dL)—**Median (IQR)	169 (132, 231)	201 (145, 290)	0.009^w^
**B-type natriuretic peptide (pg/mL)—**Median (IQR)	760 (207, 1456)	1254 (570, 2674)	0.005^w^
**Lactate (mg/dL)—**Median (IQR)	2.4 (1.5, 4.4)	4.8 (2.9, 9.9)	<0.001^w^
**Troponin I (ng/mL)—**Median (IQR)	4.3 (0.3, 47.6)	6.6 (0.6, 29.0)	0.61^w^
**pH—**Median (IQR)	7.3 (7.2, 7.4)	7.3 (7.1, 7.4)	0.012^w^
**Fraction of inspired oxygen (%)—**Median (IQR)	60 (21, 100)	100 (60, 100)	<0.001^w^
**Echocardiographic assessment at shock onset**			
**LV ejection fraction (%)—**Median (IQR)	35 (21, 44)	30 (23, 40)	0.67^w^
**LV end-diastolic diameter (cm)—**Median (IQR)	4.7 (4.3, 5.4)	4.9 (4.4, 5.5)	0.53^w^
**RV systolic function—**Hyperdynamic/Normal/Low Normal/Mildly decreased	69 (79.3%)	41 (69.5%)	0.18^c^
Moderately/Severely decreased	18 (20.7%)	18 (30.5%)	—
**Tricuspid annular plane systolic excursion (mm)—**Median (IQR)	16 (12, 20)	13 (9, 16)	0.019^e^

CSWG, Cardiogenic Shock Working Group; HF, heart failure; IABP, intra-aortic balloon pump; IQR, interquartile range; LV, left ventricular; LVAD, left ventricular assist device; MCS, mechanical circulatory support; NSTEMI, non-ST-elevation myocardial infarction; RV, right ventricular; SCAI, Society for Cardiovascular Angiography & Interventions; SD, standard deviation; STEMI, ST-elevation myocardial infarction; VA-ECMO, veno-arterial extracorporeal membrane oxygenation.

^a^Only applies if previous question is Yes.

^c^Chi-squared test. ^e^Exact Wilcoxon rank sum test. ^f^Fisher’s exact test. ^t^*T*-test. ^w^Wilcoxon rank sum test.

There were no statistically significant differences between groups in the prevalence of cardiac risk factors including hypertension, diabetes mellitus, smoking, hyperlipidaemia, chronic kidney disease, or prior cardiovascular disease including a prior diagnosis of coronary artery disease, chronic HF, and valvular heart disease. Haemodynamic measurements at shock onset were comparable between groups, except for a higher cardiac output [3.8 (3.0, 5.4) vs. 3.6 (2.5, 4.2) L/min, *P* = 0.026], power output [0.8 (0.6, 1.1) vs. 0.7 (0.4, 0.8) Watts, *P* = 0.030], and index [1.9 (1.5, 2.6) vs. 1.8 (1.2, 2.0) L/min/m^2^, *P* = 0.016] as measured by thermodilution in patients achieving NHS compared to patients who died or received advanced HF therapies. Laboratory assessment at shock onset when comparing patients achieving NHS to those who died or survived post-advanced HF therapies, revealed a lower serum creatinine [1.2 (1.0, 1.6) vs. 1.5 (1.1, 2.0) mg/dL, *P* < 0.001], B-type natriuretic peptide [760 (207, 1456) vs. 1254 (570, 2674) pg/mL, *P* = 0.005], serum lactate [2.4 (1.5, 4.4) vs. 4.8 (2.9, 9.9) mg/dL, *P* < 0.001], and pH [7.3 (7.2, 7.4) vs. 7.3 (7.1, 7.4), *P* = 0.012], in patients with NHS. Last, echocardiographic assessment at shock onset was comparable except a higher tricuspid annular plane systolic excursion [16 (12, 20) vs. 13 (9, 16) mm, *P* = 0.019], in patients with NHS compared to patients who died or survived after receiving advanced HF therapies.

Data on left heart catheterization (LHC) and revascularization in patients discharged with a native heart vs. patients who died or received advanced HF therapies are presented in *Table 2*. LHC was performed in 160/168 patients achieving NHS and 138/148 patients who died or underwent advanced HF therapies (95.2 vs. 93.2%, *P* = 0.45), with comparable rates in performance of LHC at an outside hospital or the University of Utah. Patients who died or received advanced HF therapies had higher rates of left main artery disease (22.5 vs. 13.1%, *P* = 0.034), lower rates of coronary intervention (69.6 vs. 80.6%, *P* = 0.027), and a trend towards a higher number of coronary stents deployed (2.0 ± 1.2 vs. 1.7 ± 1.0, *P* = 0.07), when compared with patient who survived with a native heart.

**Table 2:** Left heart catheterization and revascularization data in patients stratified by in-hospital outcome

Variable	Native heart survival (*N* = 168)	Survival post-advanced HF therapies or death (*N* = 148)	*P*-value
**Left heart catheterization**	160 (95.2%)	138 (93.2%)	0.45^c^
**Left heart catheterization location—**Outside hospital**^a^**	57 (35.6%)	43 (31.2%)	0.42^e^
University of Utah Hospital	103 (64.4%)	95 (68.8%)	—
**Coronary artery dominance—**Codominant**^a^**	12 (8.1%)	16 (13.2%)	0.36^e^
Left	14 (9.5%)	9 (7.4%)	—
Right	122 (82.4%)	96 (79.3%)	—
**Left main artery disease^a^**	21 (13.1%)	31 (22.5%)	0.034^e^
**Left anterior descending artery disease^a^**	134 (83.8%)	117 (84.8%)	0.81^e^
**Left circumflex artery disease^a^**	90 (56.2%)	77 (55.8%)	0.94^e^
**Right coronary artery disease^a^**	97 (60.6%)	90 (65.2%)	0.41^e^
**Multivessel disease^a^**	91 (56.9%)	90 (65.2%)	0.14^e^
**Diseased vessels number—**Mean (SD)**^a^**	2.1 (1.0)	2.3 (1.1)	0.22^w^
**Culprit vessel—**Left anterior descending artery**^a^**	72 (45.0%)	47 (34.1%)	0.13^f^
Right coronary artery	34 (21.2%)	26 (18.8%)	—
Left circumflex artery	17 (10.6%)	15 (10.9%)	—
Other artery	2 (1.2%)	4 (2.9%)	—
Unidentified artery	35 (21.9%)	46 (33.3%)	—
**Coronary intervention^a^**	129 (80.6%)	96 (69.6%)	0.027^e^
**Coronary intervention type—**Bare metal stent**^b^**	2 (1.6%)	5 (5.2%)	0.23^f^
Drug-eluting stent	121 (93.8%)	89 (92.7%)	—
Balloon angioplasty or embolectomy	6 (4.7%)	2 (2.1%)	—
**Number of stents—**Mean (SD)**^b^**	1.7 (1.0)	2.0 (1.2)	0.07^w^
**Referral for coronary artery bypass graft surgery^a^**	20 (12.8%)	12 (9.0%)	0.31^e^

SD, standard deviation.

^a^Applies to patients who underwent left heart catheterization.

^b^Applies to patients who underwent coronary intervention.

^c^Chi-squared test. ^e^Exact Wilcoxon rank sum test. ^f^Fisher’s exact test. ^w^Wilcoxon rank sum test.

### Adverse events during Index hospitalization

**Figure 2:**
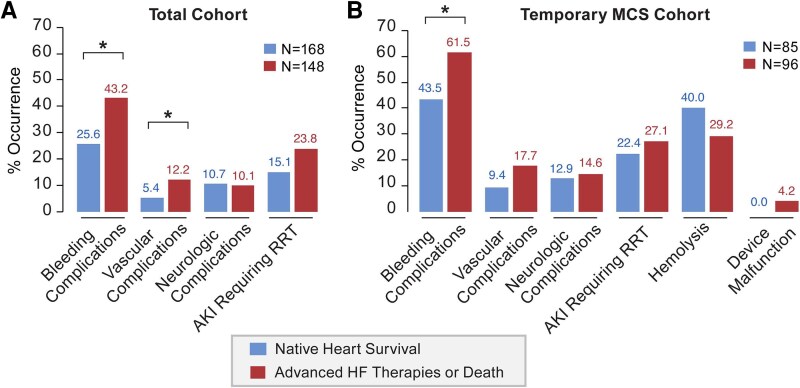
Adverse event rates during Index hospitalization. AKI, acute kidney injury; HF, heart failure; MCS, mechanical circulatory support; RRT, renal replacement therapy; * indicates statistically significant difference at *P* < 0.05.

The rates of adverse events during index hospitalization are presented in *Figure 2* and Supplementary material online, *Table S2A*. Patients achieving NHS compared to patients surviving after advanced HF or who died during index hospitalization less commonly had bleeding (25.6 vs. 43.2%, *P* < 0.001) and vascular complications (5.4 vs. 12.2%, *P* < 0.001), while neurologic complication rates were comparable between groups. AKI requiring RRT approached the statistical significance threshold, with lower rates in patients discharged with a native heart compared to patients who underwent advanced HF therapies or died (15.1 vs. 23.8%, *P* = 0.050). The rates of adverse events in the cohort of patients undergoing temporary MCS is presented in *Figure 2* and Supplementary material online, *Table S2B*. Patients who died or survived the index hospitalization after advanced HF therapies had higher rates of bleeding complications compared to patients who survived with a native heart (61.5 vs. 43.5%, *P* = 0.016). Vascular and neurologic complications, AKI requiring RRT, haemolysis, and device malfunction rates were comparable between groups.

### Survival, Major cardiac interventions, and hospital readmissions within 1-year following Index hospitalization discharge

**Figure 3:**
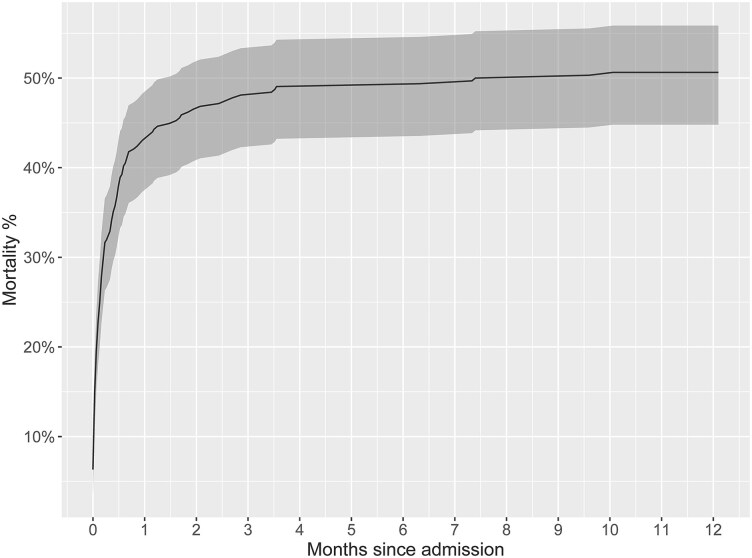
All-cause mortality up to 1-year following cardiogenic shock presentation.

Out of 316 patients suffering AMI-CS, 176 (55.7%) survived the index hospitalization, 170 (53.8%) were alive 30 days post-discharge, and 156 (49.4%) were alive 1-year post-discharge (*Table 3*), with the Kaplan–Meier mortality curve presented in *Figure 3*. Overall survival was statistically significantly different across SCAI stages at CS onset (*P* = 0.044) as evident in Supplementary material online, *Figure S1*. When comparing patients with SCAI stage B, C, D, and E, survival was 65.2, 67.9, 56.1, and 47.3% at discharge, 65.2, 65.4, 53.0, and 45.9% at 30 days post-discharge, and 60.9, 59.3, 48.5, and 42.5% at 1-year post-discharge, respectively. Overall survival was comparable when stratifying patients based on MI location (left anterior descending artery vs. other coronary artery; *P* = 0.79) as evident in Supplementary material online, *Figure S2*. Of 316 patients, 246 (77.8%) underwent revascularization, with 222 (90.2%) percutaneous coronary intervention, 18 (7.3%) coronary artery bypass graft surgery, and 6 (2.4%) both, 11 (3.5%) underwent valve repair or replacement, 10 (3.2%) LVAD implantation, 3 (0.9%) temporary surgical right-sided VAD implantation, and 3 (0.9%) HTx by 1-year post-index hospitalization discharge (*Table 3*). Of 176 patients who survived the index hospitalization, 56 (31.8%) had at least one hospital readmission for all reasons, and 30 (17.0%) one HF-related readmission by 1-year post-discharge (*Table 3*).

**Table 3:** Survival, major cardiac interventions, and hospital readmissions within 1 year following index hospitalization discharge

Outcomes	*N* (%)
**Survival**	**All patients (*N*** **=** **316)**
Survival to hospital discharge	176 (55.7%)
Survival 30 days post-hospital discharge	170 (53.8%)
Survival 1-year post-hospital discharge	156 (49.4%)
**Major cardiac interventions**	**All patients (*N*** **=** **316)**
Revascularization	246 (77.8%)
Percutaneous coronary intervention^a^	222 (90.2%)
Coronary artery bypass graft surgery^a^	18 (7.3%)
Both percutaneous coronary intervention and coronary artery bypass graft surgery^a^	6 (2.4%)
Valve repair/replacement	11 (3.5%)
Surgical^a^	8 (72.7%)
Mitral valve^b^	4 (50.0%)
Aortic valve^b^	3 (25.0%)
Multiple valves^b^	2 (25.0%)
Percutaneous^a^	3 (27.3%)
Aortic valve^a^	3 (100%)
Durable LVAD implantation	10 (3.2%)
Temporary surgical RVAD implantation	3 (0.9%)
Heart transplantation	3 (0.9%)
**Hospital readmissions**	**Survivors to hospital discharge (*N*** **=** **176)**
All-reason readmissions	56 (31.8%)
Recurrent all-reason readmissions^a^	26 (46.4%)
Heart failure-related readmissions	30 (17.0%)
Recurrent heart failure-related readmissions^a^	14 (46.7%)

LVAD, left ventricular assist device; RVAD, right ventricular assist device.

^a^Only applies if previous question is Yes.

^b^Only applies if Valve repair/replacement is Surgical or Percutaneous.

### Clinical characteristics at shock onset associated with the likelihood of NHS

Univariable logistic regression applied to all available clinical variables is presented in Supplementary material online, *Table S3*. Demographics, medical and social history, and laboratory, haemodynamic, and imaging data collected at shock onset were considered for inclusion in a multivariable model assessing the likelihood for NHS (*Table 4*, **Multivariable Model 1**). The following clinical variables were statistically significantly associated with NHS likelihood, after multivariable adjustments: age per 10 years increase [OR: 0.72 (0.59–0.89), *P* = 0.003], VIS [OR: 0.98 (0.97–1.00), *P* = 0.006), serum creatinine [OR: 0.77 (0.60–0.99), *P* = 0.043], serum lactate (Log) [OR: 0.57 (0.41–0.79), *P* < 0.001], coronary intervention [OR: 3.10 (1.42–6.75), *P* = 0.005], and number of stents deployed per unit increase [OR: 0.74 (0.56–0.97), *P* = 0.028].

**Table 4:** Multivariable assessment of clinical characteristics associated with the likelihood of native heart survival

	Univariable analysis		Multivariable model 1		Multivariable model 2	
Variable	OR (95% CI)	*P*-value	OR (95% CI)	*P*-value	OR (95% CI)	*P*-value
**Clinical variables at shock onset**						
Age (per 10 years)	0.75 (0.63,0.91)	**0**.**003**	0.72 (0.59,0.89)	**0.003**	0.60 (0.47,0.76)	**<0**.**001**
Cardiac arrest (yes vs. no)	0.64 (0.40,1.02)	0.06	—	—	—	—
Vasoactive Inotropic Score at shock onset	0.98 (0.97,0.99)	**0**.**002**	0.98 (0.97,1.00)	**0.006**	0.99 (0.97,1.00)	**0**.**029**
Pulmonary artery pulsatility index (Log)	1.10 (0.87,1.38)	0.44	1.11 (0.84,1.45)	0.47	1.02 (0.73,1.42)	0.90
Pulmonary capillary wedge pressure (mmHg)	0.97 (0.93,1.01)	0.21	0.98 (0.94,1.03)	0.51	0.99 (0.94,1.04)	0.59
Haemoglobin (g/dL)	1.07 (0.99,1.16)	0.09	—	—	—	—
Serum creatinine (mg/dL)	0.71 (0.56,0.88)	**0**.**003**	0.77 (0.60,0.99)	**0.043**	0.70 (0.53,0.93)	**0**.**014**
Alanine transaminase (mg/dL) (per 1000 increase)	0.63 (0.36,1.10)	0.11	—	—	—	—
Lactate (mg/dL) (Log)	0.58 (0.43,0.78)	**<0**.**001**	0.57 (0.41,0.79)	**<0.001**	0.70 (0.48,1.02)	0.07
pH (per unit increase)	4.12 (0.80,21.12)	0.09	—	—	—	—
Left main disease (yes vs. no)	0.56 (0.31,1.01)	0.06	0.62 (0.31,1.23)	0.17	0.58 (0.27,1.21)	0.15
Multivessel disease (yes vs. no)	0.70 (0.44,1.12)	0.14	—	—	—	—
Culprit vessel (unknown vs. known)	0.61 (0.36,1.03)	0.06	—	—	—	—
Coronary intervention (yes vs. no)^a^	2.94 (1.50,5.76)	**0**.**002**	3.10 (1.42,6.75)	**0.005**	3.00 (1.29,7.00)	**0**.**012**
Number of stents (per unit increase)^a^	0.77 (0.60,0.98)	**0**.**034**	0.74 (0.56,0.97)	**0.028**	0.74 (0.55,0.99)	**0**.**041**
**Clinical variables associated with overall management during shock hospitalization**						
Temporary MCS deployment (OSH vs. no MCS)^b^	0.73 (0.41,1.29)	0.28	—	—	1.20 (0.43,3.34)	0.73
Temporary MCS deployment (University of Utah vs. no MCS)^b^	0.50 (0.29,0.86)	**0**.**012**	—	—	0.84 (0.38,1.87)	0.67
Temporary MCS deployment (University of Utah vs. OSH)^b^	0.69 (0.37,1.26)	0.22	—	—	0.70 (0.30,1.63)	0.41
Time from shock onset to first temporary MCS (per minute increase)^b^	1.00 (0.99,1.01)	0.36	—	—	1.00 (0.98,1.01)	0.47
Escalation from intra-aortic balloon pump (yes vs. no)	0.41 (0.17,0.99)	**0**.**049**	—	—	0.44 (0.16,1.26)	0.13
Percutaneous ventricular assist device (yes vs. no)	0.51 (0.32,0.81)	**0**.**005**	—	—	0.61 (0.29,1.30)	0.20
Veno-arterial extracorporeal membrane oxygenation (yes vs. no)	0.31 (0.17,0.56)	**<0**.**001**	—	—	0.40 (0.18,0.88)	**0**.**023**
Intubation (yes vs. no)	0.20 (0.11,0.37)	**<0**.**001**	—	—	0.27 (0.13,0.57)	**<0**.**001**
Bleeding or vascular complications (yes vs. no)	0.44 (0.27,0.71)	**<0**.**001**	—	—	—	—
Acute kidney injury requiring renal replacement therapy (yes vs. no)	0.61 (0.35,1.07)	0.09	—	—	—	—

CI, confidence interval; LV, left ventricular; MCS, mechanical circulatory support; OR, odds ratio; OSH, outside hospital; RV, right ventricular.

^a^Intervention (yes vs. no) and Number of stents (per unit increase) are considered one variable, with the reference level being ‘No coronary intervention’.

^b^Temporary MCS deployment (OSH vs. no MCS, University of Utah vs. no MCS, University of Utah vs. OSH) and Time from shock onset to 1st temporary MCS (per minute increase) are considered one variable.

### Clinical characteristics during the shock hospitalization associated with the likelihood of NHS

Beyond clinical data at shock onset, we evaluated clinical variables encompassing the entire index CS hospitalization, which were added to Multivariable Model 1 (discussed above). The same clinical variables were statistically significantly associated with NHS likelihood, with the removal of serum lactate, and the addition of VA-ECMO deployment [OR: 0.40 (0.18–0.88), *P* = 0.023] and intubation [OR: 0.27 (0.13–0.57), *P* < 0.001] (*Table 4*, **Multivariable Model 2**).

## Discussion

We evaluated a contemporary, unselected cohort of patients with AMI-CS managed at a referral quaternary medical centre serving a large catchment area spanning the Mountain West region of the USA (*Figure 1*). We assessed both patients who presented directly or were transferred from outside institutions and captured clinical data pertaining to both outside and referral institution management and up to 1 year following hospital discharge. Strengths of our study include (i) high rates of temporary MCS use, (ii) inclusion of consecutive patients across a large geographic catchment area, (iii) incorporation of management practices from both outside and referral institutions, and (iv) comprehensive follow-up up to 1-year post-discharge.

CS complicates the early course of 5–10% of patients suffering AMI.^1,10^ Early revascularization was shown to confer a survival benefit for patients with AMI in the late 1990s with the landmark SHOCK trial.^9^ Since then and until recently, the immediate revascularization of the culprit coronary artery was the only treatment for AMI-CS that was shown to improve outcomes in a randomized study.^43^ Various management strategies have been implemented in the management of patients with CS including multidisciplinary shock teams and regionalized systems of care,^23,44–48^ as well as deployment of temporary MCS devices; however, survival rates remain dismal with ∼40% of patients dying at 30 days and ∼50% at 1 year.^1,10–12^ After multiple studies failing to support the use of temporary MCS in the management of AMI-CS,^12–18^ the DanGer Shock randomized trial showcased a survival benefit with the use of pVAD, with a reduction of 180-day mortality from 58.5% to 45.8% compared to standard of care.^19^ The generalizability of the trial findings has been questioned, with a recent study suggesting that two-thirds of contemporary patients with AMI-CS managed in cardiac intensive care units in North America would not meet the inclusion criteria of the study.^20^ The high incidence of temporary MCS-related adverse events also raises concerns, with implications to patient outcomes, clinical practice, and systems of care.^21,22^ In this context, we assessed consecutive, unselected patients and captured clinical data pertaining to both outside and referral institution management. Our study design allowed us to evaluate the entire disease trajectory and incorporate diverse management strategies employed at institutions of varying levels of care. Prior studies have been limited by not recording clinical data pertaining to the management of patients during management at an outside facility, with time zero being the admission to the referral centre.

Our study cohort comprised 316 patients with AMI-CS with high rates of temporary MCS utilization (181 patients, 57.3%). Our main findings can be viewed in the **Graphical Abstract**. Regarding index hospitalization outcomes, 168 patients (53.2%) achieved NHS, 140 (44.3%) died, and 8 (2.5%) were discharged after receiving advanced HF therapies. In contrast to HF-related CS where advanced HF therapy rates approach 10% of patients, the respective rate in our AMI-CS patient cohort was lower, consistent with previous reports.^7,8^ Overall survival rates were 55.7% at hospital discharge, 53.8% at 30 days, and 49.4% at 1 year, which are concordant with prior studies.^1,10–12^ From 176 patients surviving to hospital discharge, 56 (31.8%) had at least one hospital readmission and 30 (17.0%), at least one HF-related readmission within 1-year post-discharge, showcasing the importance of longer-term follow-up.

Patients discharged with a native heart compared to patients who received advanced HF therapies or died, were younger, had better end-organ function as evidenced by serum creatinine, lactate levels, and pH, haemodynamic profile and biventricular function as evidenced by B-type natriuretic peptide levels, cardiac output, power output, and index by thermodilution, tricuspid annular plane systolic excursion, and CS severity as evidenced by SCAI-CSWG stage. Regarding management, patients achieving NHS less commonly required intubation, had lower VIS at shock onset, and less frequently required temporary MCS. Notably, they had a shorter time from shock onset to temporary MCS deployment, and regarding specific temporary MCS modalities, they less commonly required escalation from IABP, required pVAD, or VA-ECMO, compared to patients who either died or required advanced HF therapies. In terms of coronary diagnostics and interventions, patients who received advanced HF therapies or died more commonly had left main artery disease, and less commonly had a successful coronary intervention compared to survivors with native heart.

Clinical factors associated with a higher likelihood of NHS, after multivariable adjustments, included demographics (younger age), end-organ function (lower serum creatinine and lactate levels at shock onset) and select management strategies (lower VIS at shock onset, successful coronary intervention with fewer stents deployed, absence of intubation/mechanical ventilation and use of VA-ECMO). Our findings are in agreement with previous studies on CS survival.^6–8,34–40,44,49^ Although factors like age are non-modifiable, indices of disease severity and management strategies, including serum creatinine and lactate at shock onset, escalation of inotropic and vasoactive support, successful coronary intervention, intubation, and use of VA-ECMO, might inform patient care with potential earlier transfer to an advanced CS centre and earlier escalation to temporary MCS as opposed to intensification of inotropic/vasoactive agents.

Early identification of CS and implementation of MCS, as opposed to escalation of pharmacotherapy, have been suggested to affect outcomes.^7,23,44,48,50–53^ Deploying a pVAD within 1.25 h from shock onset improved in-hospital survival for patients suffering AMI-CS.^51^ In a study including patients with both AMI-CS and HF-CS, mortality rates were higher with delaying temporary MCS implementation.^48^ In our study, the time from shock onset to temporary MCS deployment was shorter in patients achieving NHS, however this variable was not ultimately included in our multivariable models. Moreover, at a multivariable level the location of first temporary MCS deployment (outside institution vs. University of Utah Hospital) was not statistically significantly associated with NHS likelihood. Higher VIS at shock onset, however, was independently associated with lower NHS likelihood. Although, this observation might be influenced by the timing of transfer to our centre, earlier temporary MCS deployment as opposed to escalating inotrope/vasopressor agents, should be further explored as a potential intervention target in the management of patients suffering AMI-CS.

In terms of adverse events during CS hospitalization, bleeding and vascular complications were more common in patients who died or received advanced HF therapies compared to patients achieving NHS. The observation that bleeding and vascular complications were more frequent among patients requiring temporary MCS highlights the ongoing balance between the potential benefits and risks of device-based support. Comparing AKI requiring RRT rates between patients who died or underwent advanced HF therapies and those who survived with a native heart approached statistical significance (*P* = 0.050) and were lower in the latter group. Whether adverse event rates are associated with more severe disease and need for MCS or mediate the lower rates of NHS warrants further investigation.

## Limitations

Besides the prospective enrolment of patients, the review of the electronic medical records and the retrospective collection of clinical information makes our study prone to inherent limitations of observational studies, including unmeasured confounding and selection bias. Most patients in our study were male (73.4%) and white (87.0%), which limits the applicability of our findings to women and non-white races. The limited sample size and the presentation or transfer of patients to a quaternary healthcare institution might limit the generalizability of our findings. The inclusion, however, of patients initially presenting and developing shock at outside facilities, the collection of clinical data during this timeframe, and thus the incorporation of variations in clinical practices, makes our study more comprehensive and generalizable. Nonetheless, non-uniformity and missingness in outside clinical data recording should be acknowledged. Total ischaemic time which is particularly relevant for patients with ST-segment elevation MI was not consistently available. Defining the exact time of shock onset is challenging besides the use of a common and widely accepted definition. Finally, we might have included patients ineligible for advanced HF therapies with a potential impact on our findings.

## Conclusion

We assessed consecutive, unselected patients suffering AMI-CS who presented or were transferred to our institution, captured their complete disease trajectory including outside facility stay, and identified clinical factors associated with a higher likelihood of NHS. Although factors like age are non-modifiable, parameters reflecting disease severity, end-organ function, and management including serum creatinine and lactate levels, level of vasoactive/inotropic support, successful coronary intervention, intubation, and use of VA-ECMO, might inform care practices for patients with AMI-CS. In the setting of increasing temporary MCS utilization, earlier transfer to an advanced CS centre and earlier implementation of MCS as opposed to escalation of pharmacotherapy, warrant further investigation as potential management targets. The impact and management of adverse events including bleeding and vascular complications, and renal impairment, should be evaluated in prospective studies, especially with increasing MCS use. Last, high readmission rates, with 31.8% of patients having at least one hospital readmission overall and 17.0% having at least one HF-related readmission within 1-year post-discharge, showcase the importance of longer-term patient follow-up.

## Supplementary Material

oeag015_Supplementary_Data

## Data Availability

The data and analytic methods of the study will be made available from the corresponding author upon reasonable request.
