# Supplementary material for: Factors associated with native heart survival and intermediate-term outcomes in acute myocardial infarction-related cardiogenic shock
Source: Eur Heart J Open. 2026 Feb 26;6(1):oeag015. doi: 10.1093/ehjopen/oeag015 (PMC12941215; doi:10.1093/ehjopen/oeag015)
Supplement: oeag015_Supplementary_Data [file oeag015_supplementary_data.docx]

**SUPPLEMENTAL MATERIAL**

Tables S1-S3, Figures S1-S2

**Table S1.** Baseline Clinical Characteristics in Patients Stratified by In-Hospital Outcome

*CSWG: Cardiogenic Shock Working Group, HF: heart failure, IABP: intra-aortic balloon pump, ICM: ischemic cardiomyopathy, IQR: interquartile range, LV: left ventricular, LVAD: left ventricular assist device, MCS: mechanical circulatory support, NICM: non-ischemic cardiomyopathy, NSTEMI: non-ST-elevation myocardial infarction, RV: right ventricular, SCAI: Society for Cardiovascular Angiography & Interventions, SD: standard deviation, STEMI: ST-elevation myocardial infarction, VA-ECMO: veno-arterial extracorporeal membrane oxygenation, VAD: ventricular assist device*

| **Variable** | **Native Heart Survival   (N=168)** | **Survival Post-Advanced HF Therapies or Death   (N=148)** | **P-value** | **Number of Missing Values** | **Percentage of Missing Values** |
| --- | --- | --- | --- | --- | --- |
| **Demographics** |  | | | | |
| **Age (years) -** Mean (SD) | 62 (12) | 66 (13) | 0.002^t^ | 0 | 0% |
| **Sex -** Female | 44 (26.2%) | 40 (27.0%) | 0.87^c^ | 0 | 0% |
| Male | 124 (73.8%) | 108 (73.0%) | - | - | - |
| **Race -** White | 140 (87.0%) | 120 (87.0%) | 0.36^f^ | 17 | 5.4% |
| Black/African American | 1 (0.6%) | 1 (0.7%) | - | - | - |
| American Indian/Alaska Native | 4 (2.5%) | 3 (2.2%) | - | - | - |
| Native Hawaiian/Other Pacific Islander | 0 (0.0%) | 4 (2.9%) | - | - | - |
| Asian | 3 (1.9%) | 2 (1.4%) | - | - | - |
| Other | 13 (8.1%) | 8 (5.8%) | - | - | - |
| **Ethnicity -** Hispanic/Latino | 13 (8.0%) | 8 (6.4%) | 0.60^c^ | 29 | 9.2% |
| Not Hispanic/Latino | 149 (92.0%) | 117 (93.6%) | - | - | - |
| **Body mass index (kg/m^2^) –** Median (IQR) | 29 (26, 32) | 29 (26, 33) | 0.86^w^ | 5 | 1.6% |
| **Cardiogenic Shock Management and Treatment** |  | | | | |
| **Transfer from outside hospital** | 122 (72.6%) | 104 (70.3%) | 0.64^c^ | 0 | 0% |
| **Shock onset hospital -** University of Utah Hospital | 100 (59.5%) | 88 (59.5%) | 0.99^c^ | 0 | 0% |
| Outside hospital | 68 (40.5%) | 60 (40.5%) | - | - | - |
| **University of Utah Hospital length of stay (days) -** Median (IQR) | 10 (7, 20) | 4 (1, 14) | <0.001^w^ | 0 |  |
| **Outside hospital length of stay (days) -** Median (IQR) | 1 (0, 2) | 1 (0, 2) | 0.83^w^ | 5 | 1.6% |
| **Chronicity of disease –** Acute heart failure | 149 (88.7%) | 134 (90.5%) | 0.59^c^ | 0 | 0% |
| Acute-on-chronic heart failure | 19 (11.3%) | 14 (9.5%) | - | - | - |
| **Acute coronary syndrome type -** NSTEMI | 49 (29.2%) | 51 (34.5%) | 0.31^c^ | 0 | 0% |
| STEMI | 119 (70.8%) | 97 (65.5%) | - | - | - |
| **Acute coronary syndrome complication** | 6 (3.6%) | 5 (3.4%) | 0.93^c^ | 0 | 0% |
| ***Acute coronary syndrome complication type –** Papillary muscle rupture | 1 (16.7%) | 0 (0.0%) | 1.00^f^ | 0 | 0% |
| Ventricular septal defect | 5 (83.3%) | 5 (100%) | - | - | - |
| **SCAI-CSWG stage at shock onset -** B | 14 (8.3%) | 9 (6.1%) | 0.035^c^ | 0 | 0% |
| C | 53 (31.5%) | 28 (18.9%) | - | - | - |
| D | 34 (20.2%) | 32 (21.6%) | - | - | - |
| E | 67 (39.9%) | 79 (53.4%) | - | - | - |
| **Cardiac arrest** | 52 (31.0%) | 61 (41.2%) | 0.06^c^ | 0 | 0% |
| **^*^In-Hospital** | 22 (42.3%) | 31 (50.8%) | 0.37^c^ | 0 | 0% |
| **^*^In-Hospital-Initial shockable rhythm** | 12 (54.5%) | 21 (67.7%) | 0.11^f^ | 0 | 0% |
| **^*^Out-of-Hospital** | 31 (59.6%) | 32 (52.5%) | 0.45^c^ | 0 | 0% |
| **^*^Out-of-Hospital-Initial shockable rhythm** | 27 (87.1%) | 24 (75.0%) | 0.44^f^ | 0 | 0% |
| **Inotropes/Vasopressors at shock onset** | 104 (61.9%) | 91 (61.5%) | 0.94^c^ | 0 | 0% |
| **Inotrope/Vasopressors number -** Median (IQR) | 1 (0, 2) | 2 (0, 3) | 0.26^w^ | 0 | 0% |
| **Vasoactive Inotropic Score -** Median (IQR) | 4.2 (0.0, 14.3) | 9.1 (0.0, 21.8) | 0.08^w^ | 0 | 0% |
| **Pulmonary artery catheter placement** | 121 (72.0%) | 110 (74.3%) | 0.65^c^ | 0 | 0% |
| **Intubation** | 105 (62.5%) | 132 (89.2%) | <0.001^c^ | 0 | 0% |
| **Temporary MCS** | 85 (50.6%) | 96 (64.9%) | 0.011^c^ | 0 | 0% |
| **^*^Time to 1st temporary MCS (min) -** Median (IQR) | 90 (30, 270) | 152 (60, 511) | 0.009^w^ | 0 | 0% |
| ***Temporary MCS implant at outside hospital** | 40 (47.1%) | 35 (36.5%) | 0.15^c^ | 0 | 0% |
| **IABP** | 44 (26.2%) | 34 (23.0%) | 0.51^c^ | 0 | 0% |
| **^*^IABP access site -** Femoral | 43 (100%) | 33 (97.1%) | 0.44^f^ | 0 | 0% |
| Femoral, Axillary | 0 (0%) | 1 (2.9%) | - | - | - |
| ***Escalation from IABP** | 8 (18.2%) | 16 (47.1%) | 0.006^c^ | 0 | 0% |
| **Impella** | 45 (26.8%) | 62 (41.9%) | 0.005^c^ | 0 | 0% |
| **^*^Impella Type** - CP | 34 (75.6%) | 56 (90.3%) | 0.08^f^ | 0 | 0% |
| RP | 4 (8.9%) | 3 (4.8%) | - | - | - |
| 2.5 | 4 (8.9%) | 0 (0%) | - | - | - |
| 5.0 | 2 (4.4%) | 1 (1.6%) | - | - | - |
| Multiple Impella devices | 1 (2.2%) | 2 (3.2%) | - | - | - |
| ***Escalation from Impella** | 10 (22.2%) | 14 (22.6%) | 0.97^c^ | 0 | 0% |
| **VA-ECMO** | 19 (11.3%) | 43 (29.1%) | <0.001^c^ | 0 | 0% |
| **^*^VA-ECMO venting -** IABP | 1 (5.3%) | 4 (9.3%) | 1.00^f^ | 0 | 0% |
| Impella | 11 (57.9%) | 25 (58.1%) | - | - | - |
| No | 7 (36.8%) | 14 (32.6%) | - | - | - |
| **Temporary surgical VAD** | 2 (1.2%) | 6 (4.1%) | 0.15^f^ | 0 | 0% |
| **ProtekDuo** | 1 (0.6%) | 0 (0%) | 1.00^f^ | 0 | 0% |
| **Heart transplantation** | 0 (0%) | 0 (0%) | 1.00^f^ | 0 | 0% |
| **Durable LVAD implantation** | 0 (0%) | 9 (6.1%) | <0.001^f^ | 0 | 0% |
| **Past Medical History** |  | | | | |
| **Hypertension** | 108 (64.3%) | 100 (69.9%) | 0.29^c^ | 5 | 1.6% |
| **Diabetes mellitus** | 55 (32.7%) | 56 (39.2%) | 0.24^c^ | 5 | 1.6% |
| **Smoking -** Current smoker | 43 (25.7%) | 24 (16.9%) | 0.09^c^ | 7 | 2.2% |
| Former smoker | 44 (26.3%) | 34 (23.9%) | - | - | - |
| Non-smoker | 80 (47.9%) | 84 (59.2%) | - | - | - |
| **Hyperlipidemia** | 77 (45.8%) | 75 (52.4%) | 0.24^c^ | 5 | 1.6% |
| **Chronic Kidney Disease Stage III-V** | 13 (7.7%) | 18 (12.6%) | 0.15^c^ | 5 | 1.6% |
| **^*^Dialysis** | 3 (23.1%) | 5 (27.8%) | 1.00^f^ | 0 | 0% |
| **Coronary artery disease** | 60 (35.7%) | 57 (39.9%) | 0.45^c^ | 5 | 1.6% |
| **^*^Prior coronary artery bypass graft surgery** | 13 (21.7%) | 17 (29.8%) | 0.31^c^ | 0 | 0% |
| **^*^Prior percutaneous coronary intervention** | 34 (56.7%) | 32 (56.1%) | 0.95^c^ | 0 | 0% |
| **Prior myocardial infarction** | 35 (20.8%) | 37 (25.9%) | 0.29^c^ | 0 | 0% |
| **Chronic heart failure** | 19 (11.3%) | 14 (9.5%) | 0.59^c^ | 0 | 0% |
| **^*^Heart failure etiology** - ICM | 15 (78.9%) | 13 (92.9%) | 0.78^f^ | 0 | 0% |
| NICM | 3 (15.8%) | 1 (7.1%) | - | - | - |
| Combined ICM/NICM | 1 (5.3%) | 0 (0%) | - | - | - |
| **^*^Reduced LV ejection fraction (<50%)** | 17 (89.5%) | 13 (92.9%) | 1.00^f^ | 0 | 0% |
| **^*^New York Heart Association class -** I | 1 (6.2%) | 0 (0%) | 0.59^f^ | 7 | 2.2% |
| II | 5 (31.2%) | 1 (10.0%) | - | - | - |
| III | 7 (43.8%) | 6 (60.0%) | - | - | - |
| IV | 3 (18.8%) | 3 (30.0%) | - | - | - |
| **Valvular heart disease** | 3 (1.8%) | 5 (3.5%) | 0.48^f^ | 5 | 1.6% |
| **Prior valve replacement/repair** | 0 (0%) | 3 (2.1%) | 0.10^f^ | 13 | 4.1% |
| **Hemodynamics at Shock Onset** |  | | | | |
| **Heart rate (beats per minute) -** Median (IQR) | 93 (80, 107) | 90 (76, 108) | 0.41^w^ | 76 | 24.1% |
| **Systolic arterial blood pressure (mmHg) -** Median (IQR) | 111 (97, 127) | 107 (93, 125) | 0.37^w^ | 76 | 24.1% |
| **Diastolic arterial blood pressure (mmHg) -** Median (IQR) | 70 (60, 83) | 70 (60, 82) | 0.66^w^ | 76 | 24.1% |
| **Mean arterial blood pressure (mmHg) -** Median (IQR) | 84 (75, 97) | 82 (73, 94) | 0.52^w^ | 76 | 24.1% |
| **Mean right atrial pressure (mmHg) -** Median (IQR) | 13 (9, 16) | 13 (11, 19) | 0.08^w^ | 137 | 43.4% |
| **Pulmonary artery systolic pressure (mmHg) -** Median (IQR) | 41 (33, 51) | 44 (33, 56) | 0.35^w^ | 149 | 47.2% |
| **Pulmonary artery diastolic pressure (mmHg) -** Median (IQR) | 23 (17, 30) | 27 (21, 32) | 0.14^w^ | 150 | 47.5% |
| **Pulmonary artery mean pressure (mmHg) -** Median (IQR) | 30 (25, 39) | 34 (25, 41) | 0.17^w^ | 151 | 47.8% |
| **Pulmonary capillary wedge pressure (mmHg) -** Median (IQR) | 21 (17, 27) | 24 (18, 30) | 0.06^w^ | 153 | 48.4% |
| **Pulmonary artery pulsatility index -** Median (IQR) | 1.4 (1.0, 2.0) | 1.1 (0.8, 1.9) | 0.10^w^ | 157 | 49.7% |
| **Aortic pulsatility index -** Median (IQR) | 1.9 (1.3, 2.8) | 1.7 (1.1, 2.2) | 0.17^w^ | 210 | 66.5% |
| **Cardiac output by Fick (L/min) -** Median (IQR) | 3.9 (3.1, 5.5) | 3.9 (2.9, 4.7) | 0.75^w^ | 181 | 57.3% |
| **Cardiac power output by Fick (Watts) -** Median (IQR) | 0.7 (0.6, 1.0) | 0.7 (0.6, 1.0) | 0.84^e^ | 230 | 72.8% |
| **Cardiac index by Fick (L/min/m^2^) -** Median (IQR) | 1.9 (1.6, 2.5) | 2.0 (1.5, 2.4) | 0.84^w^ | 181 | 57.3% |
| **Cardiac output by thermodilution (L/min) -** Median (IQR) | 3.8 (3.0, 5.4) | 3.6 (2.5, 4.2) | 0.026^w^ | 181 | 57.3% |
| **Cardiac power output by thermodilution (Watts) -** Median (IQR) | 0.8 (0.6, 1.1) | 0.7 (0.4, 0.8) | 0.030^e^ | 230 | 72.8% |
| **Cardiac index by thermodilution (L/min/m^2^)-** Median (IQR) | 1.9 (1.5, 2.6) | 1.8 (1.2, 2.0) | 0.016^w^ | 181 | 57.3% |
| **Systemic vascular resistance (dynes*s/cm^5^) -** Median (IQR) | 1284 (873, 1659) | 1327 (848, 1609) | 0.78^w^ | 177 | 56.0% |
| **Pulmonary vascular resistance (dynes*s/cm^5^) -** Median (IQR) | 182 (102, 257) | 157 (105, 334) | 0.92^w^ | 177 | 56.0% |
| **Laboratory Assessment at Shock Onset** |  | | | | |
| **Hemoglobin (g/dL) -** Mean (SD) | 13.6 (2.7) | 13.0 (3.1) | 0.09^t^ | 28 | 8.9% |
| **Serum creatinine (mg/dL) -** Median (IQR) | 1.2 (1.0, 1.6) | 1.5 (1.1, 2.0) | <0.001^w^ | 27 | 8.5% |
| **Aspartate transaminase (mg/dL) -** Median (IQR) | 110 (35, 235) | 114 (47, 342) | 0.09^w^ | 51 | 16.1% |
| **Alanine transaminase (mg/dL) -** Median (IQR) | 52 (29, 121) | 68 (30, 207) | 0.13^w^ | 51 | 16.1% |
| **Total bilirubin (mg/dL) -** Median (IQR) | 0.8 (0.5, 1.1) | 0.7 (0.5, 1.1) | 0.41^w^ | 52 | 16.5% |
| **Blood glucose (mg/dL) -** Median (IQR) | 169 (132, 231) | 201 (145, 290) | 0.009^w^ | 28 | 8.9% |
| **B-type natriuretic peptide (pg/mL) -** Median (IQR) | 760 (207, 1456) | 1254 (570, 2674) | 0.005^w^ | 180 | 57.0% |
| **Lactate (mg/dL) -** Median (IQR) | 2.4 (1.5, 4.4) | 4.8 (2.9, 9.9) | <0.001^w^ | 95 | 30.1% |
| **Troponin I (ng/mL) -** Median (IQR) | 4.3 (0.3, 47.6) | 6.6 (0.6, 29.0) | 0.61^w^ | 50 | 15.8% |
| **Lactate dehydrogenase (IU/L) -** Median (IQR) | 809 (512, 1238) | 1143 (537, 1663) | 0.22^e^ | 252 | 79.7% |
| **pH -** Median (IQR) | 7.3 (7.2, 7.4) | 7.3 (7.1, 7.4) | 0.012^w^ | 92 | 29.1% |
| **Bicarbonate (mmol/L) -** Mean (SD) | 19 (5) | 18 (6) | 0.08^t^ | 95 | 30.1% |
| **Arterial partial pressure of oxygen (mmHg) -** Median (IQR) | 82.4 (46.3, 133.0) | 72.7 (44.0, 118.5) | 0.29^w^ | 94 | 29.7% |
| **Fraction of inspired oxygen (%) -** Median (IQR) | 60 (21, 100) | 100 (60, 100) | <0.001^w^ | 127 | 40.2% |
| **Echocardiographic Assessment at Shock Onset** |  | | | | |
| **LV ejection fraction (%) -** Median (IQR) | 35 (21, 44) | 30 (23, 40) | 0.67^w^ | 111 | 35.1% |
| **LV end-diastolic diameter (cm) -** Median (IQR) | 4.7 (4.3, 5.4) | 4.9 (4.4, 5.5) | 0.53^w^ | 154 | 48.7% |
| **Aortic valve regurgitation -** None/Trivial/Mild | 93 (97.9%) | 62 (96.9%) | 1.00^f^ | 157 | 49.7% |
| Moderate/Severe | 2 (2.1%) | 2 (3.1%) | - | - | - |
| **Mitral valve regurgitation -** None/Trivial/Mild | 87 (85.3%) | 75 (93.8%) | 0.07^c^ | 134 | 42.4% |
| Moderate/Severe | 15 (14.7%) | 5 (6.2%) | - | - | - |
| **Tricuspid regurgitation -** None/Trivial/Mild | 96 (91.4%) | 67 (90.5%) | 0.84^c^ | 137 | 43.4% |
| Moderate/Severe | 9 (8.6%) | 7 (9.5%) | - | - | - |
| **RV systolic function –** Hyperdynamic/Normal/Low Normal/Mildly Decreased | 69 (79.3%) | 41 (69.5%) | 0.18^c^ | 170 | 53.8% |
| Moderately/Severely Decreased | 18 (20.7%) | 18 (30.5%) | - | - | - |
| **Tricuspid annular plane systolic excursion (mm) -** Median (IQR) | 16 (12, 20) | 13 (9, 16) | 0.019^e^ | 199 | 63.0% |

^*^ Only applies if previous question is Yes.

^a^ ANOVA, ^c^ Chi-squared test, ^e^ Exact wilcoxon rank sum test, ^f^ Fisher's exact test, ^t^ T-test, ^w^ Wilcoxon rank sum test

**Table S2.** Adverse Event Rates During Index Hospitalization

*HF: heart failure*

**a) Total Cohort**

| **Variable** | **Native Heart Survival   (N=168)** | **Survival Post-Advanced HF Therapies or Death   (N=148)** | **P-value** |
| --- | --- | --- | --- |
| **Bleeding Complications** | 43 (25.6%) | 64 (43.2%) | <0.001^c^ |
| Type 3 bleeding | 43 (25.6%) | 64 (43.2%) | <0.001^c^ |
| **Vascular Complications** | 9 (5.4%) | 18 (12.2%) | 0.031^c^ |
| Vascular Repair | 8 (4.8%) | 15 (10.1%) | 0.07^c^ |
| Amputation | 2 (1.2%) | 5 (3.4%) | 0.26^f^ |
| Fasciotomy | 6 (3.6%) | 8 (5.4%) | 0.43^c^ |
| **Neurologic Complications** | 18 (10.7%) | 15 (10.1%) | 0.87^c^ |
| Ischemic stroke | 17 (10.1%) | 14 (9.5%) | 0.84^c^ |
| Hemorrhagic stroke | 1 (0.6%) | 1 (0.7%) | 1.00^f^ |
| Transient ischemic attack | 0 (0%) | 0 (0%) | 1.00^f^ |
| **Acute Kidney Injury Requiring Renal Replacement Therapy** | 25 (15.1%) | 35 (23.8%) | 0.050^c^ |

^c^ Chi-squared test, ^f^ Fisher's exact test

**b) Temporary Mechanical Circulatory Support Cohort**

| **Variable** | **Native Heart Survival   (N=85)** | **Survival Post-Advanced HF Therapies or Death   (N=96)** | **P-value** |
| --- | --- | --- | --- |
| **Bleeding Complications** | 37 (43.5%) | 59 (61.5%) | 0.016^c^ |
| Type 3 bleeding | 37 (43.5%) | 59 (61.5%) | 0.016^c^ |
| Type 4 bleeding | 19 (22.4%) | 30 (31.2%) | 0.18^c^ |
| **Vascular Complications** | 8 (9.4%) | 17 (17.7%) | 0.11^c^ |
| Vascular Repair | 7 (8.2%) | 14 (14.6%) | 0.18^c^ |
| Amputation | 1 (1.2%) | 4 (4.2%) | 0.37^f^ |
| Fasciotomy | 5 (5.9%) | 7 (7.3%) | 0.70^c^ |
| **Neurologic Complications** | 11 (12.9%) | 14 (14.6%) | 0.75^c^ |
| Ischemic stroke | 10 (11.8%) | 13 (13.5%) | 0.72^c^ |
| Hemorrhagic stroke | 1 (1.2%) | 1 (1.0%) | 1.00^f^ |
| Transient ischemic attack | 0 (0%) | 0 (0%) | 1.00^f^ |
| **Acute Kidney Injury Requiring Renal Replacement Therapy** | 19 (22.4%) | 26 (27.1%) | 0.46^c^ |
| **Hemolysis** | 34 (40.0%) | 28 (29.2%) | 0.13^c^ |
| **Device Malfunction** | 0 (0.0%) | 4 (4.2%) | 0.12^f^ |

^c^ Chi-squared test, ^f^ Fisher's exact test

**Table S3.** Univariable logistic regression applied to clinical variables at shock onset and overall stay and management

*CI: confidence interval, CSWG: Cardiogenic Shock Working Group, IABP: intra-aortic balloon pump, ICM: ischemic cardiomyopathy, LHC: left heart catheterization, MCS: mechanical circulatory support, NICM: non-ischemic cardiomyopathy, pVAD: percutaneous ventricular assist device, SCAI: Society for Cardiovascular Angiography & Interventions, STEMI: ST-elevation myocardial infarction, VA-ECMO: veno-arterial extracorporeal membrane oxygenation*

|  | **Odds ratio (95% CI)** | **p-value** |
| --- | --- | --- |
| **Demographics** |  |  |
| Age (per 10 years) | 0.75 (0.63,0.91) | 0.003 |
| Sex (male vs. female) | 1.04 (0.63,1.72) | 0.87 |
| Race (Non-white vs. White) | 1.01 (0.52,1.97) | 0.98 |
| Ethnicity (Not Hispanic/Latino vs. Hispanic/Latino) | 1.00 (0.41,2.44) | 1.00 |
| Body mass index (per 5 kg/m^2^) | 1.00 (0.84,1.20) | 0.97 |
| **Cardiogenic Shock Management and Treatment** |  |  |
| Transfer from outside healthcare facility (yes vs. no) | 1.12 (0.69,1.83) | 0.64 |
| Shock onset hospital (University of Utah vs. outside) | 1.00 (0.64,1.57) | 0.99 |
| University of Utah Hospital length of stay (days) | 1.02 (1.00,1.04) | 0.016 |
| Outside healthcare facility length of stay (days) | 1.00 (0.93,1.09) | 0.91 |
| SCAI-CSWG stage at shock onset (C vs. B) | 1.22 (0.47,3.16) | 0.69 |
| SCAI-CSWG stage at shock onset (D vs. B) | 0.68 (0.26,1.80) | 0.44 |
| SCAI-CSWG stage at shock onset (E vs. B) | 0.55 (0.22,1.34) | 0.19 |
| Chronicity of disease (Acute-on-Chronic vs. Acute) | 1.22 (0.59,2.53) | 0.59 |
| Acute coronary syndrome type (STEMI vs. Non-STEMI) | 1.28 (0.79,2.05) | 0.31 |
| Acute coronary syndrome complication (yes vs. no) | 1.06 (0.32,3.54) | 0.93 |
| ^*^Temporary MCS (outside hospital vs. no MCS) | 0.73 (0.41,1.29) | 0.28 |
| ^*^Temporary MCS (University of Utah vs. no MCS) | 0.50 (0.29,0.86) | 0.012 |
| ^*^Temporary MCS (University of Utah vs. outside hospital) | 0.69 (0.37,1.26) | 0.22 |
| ^*^Time from shock onset to 1st temporary MCS (min) | 0.99 (0.98,1.00) | 0.26 |
| IABP (yes vs. no) | 1.19 (0.71,1.99) | 0.51 |
| Escalation from IABP (yes vs. no) | 0.41 (0.17,0.99) | 0.049 |
| pVAD (yes vs. no) | 0.51 (0.32,0.81) | 0.005 |
| Escalation from pVAD (yes vs. no) | 0.98 (0.39,2.46) | 0.97 |
| VA-ECMO (yes vs. no) | 0.31 (0.17,0.56) | <0.001 |
| Cardiac Arrest (yes vs. no) | 0.64 (0.40,1.02) | 0.06 |
| ^**^In-Hospital (yes vs. no) | 0.71 (0.34,1.49) | 0.37 |
| ^**^Out-of-Hospital (yes vs. no) | 1.34 (0.63,2.83) | 0.45 |
| Pulmonary artery catheter placement (yes vs. no) | 0.89 (0.54,1.47) | 0.65 |
| Intubation (yes vs. no) | 0.20 (0.11,0.37) | <0.001 |
| Inotropes/vasopressors at shock onset (yes vs. no) | 1.02 (0.65,1.60) | 0.94 |
| Inotropes/vasopressors number | 0.90 (0.78,1.05) | 0.18 |
| Vasoactive Inotropic Score | 0.98 (0.97,0.99) | 0.002 |
| **Past Medical History** |  |  |
| Hypertension (yes vs. no) | 0.79 (0.49,1.27) | 0.34 |
| Diabetes mellitus (yes vs. no) | 0.76 (0.48,1.21) | 0.25 |
| Smoking (former smoker vs non-smoker) | 1.30 (0.76,2.24) | 0.34 |
| Smoking (current smoker vs. non-smoker) | 1.87 (1.04,3.38) | 0.038 |
| Hyperlipidemia (yes vs. no) | 0.77 (0.49,1.21) | 0.26 |
| Chronic Kidney Disease Stage III-V (yes vs. no) | 0.59 (0.28,1.26) | 0.18 |
| Coronary artery disease (yes vs. no) | 0.85 (0.54,1.36) | 0.51 |
| Prior myocardial infarction (yes vs. no) | 0.75 (0.44,1.27) | 0.28 |
| Chronic heart failure (yes vs. no) | 1.22 (0.59,2.53) | 0.59 |
| ^**^Reduced (<50%) vs. preserved (>=50%) LV ejection fraction | 0.65 (0.05,8.02) | 0.74 |
| ^**^New York Heart Association class (III/IV vs. I/II/no heart failure) | 0.75 (0.32,1.76) | 0.50 |
| ^**^NICM vs. ICM | 2.60 (0.24,28.15) | 0.44 |
| **Hemodynamics at Shock Onset** |  |  |
| Heart rate (beats per minute) | 1.00 (0.99,1.01) | 0.73 |
| Systolic arterial pressure (mmHg) | 1.00 (0.99,1.01) | 0.86 |
| Diastolic arterial pressure (mmHg) | 1.00 (0.99,1.02) | 0.82 |
| Mean arterial pressure (mmHg) | 1.00 (0.99,1.02) | 0.87 |
| Mean right atrial pressure (mmHg) | 0.99 (0.97,1.01) | 0.32 |
| Pulmonary artery systolic pressure (mmHg) | 0.99 (0.98,1.01) | 0.49 |
| Pulmonary artery diastolic pressure (mmHg) | 0.98 (0.95,1.01) | 0.25 |
| Pulmonary artery mean pressure (mmHg) | 0.99 (0.97,1.01) | 0.39 |
| Pulmonary capillary wedge pressure (mmHg) | 0.97 (0.93,1.01) | 0.21 |
| Pulmonary artery pulsatility index (Log) | 1.10 (0.87,1.38) | 0.44 |
| Aortic pulsatility index | 1.23 (0.96,1.57) | 0.10 |
| Cardiac output by Fick (L/min) | 0.99 (0.89,1.09) | 0.78 |
| Cardiac power output by Fick (L/min) | 1.15 (0.62,2.16) | 0.66 |
| Cardiac index by Fick (L/min/m^2^) | 1.03 (0.81,1.31) | 0.83 |
| Cardiac output by thermodilution (L/min) | 1.23 (1.03,1.47) | 0.031 |
| Cardiac power output by thermodilution (L/min) | 2.15 (0.96,4.82) | 0.07 |
| Cardiac index by thermodilution (L/min/m^2^) | 1.32 (1.01,1.73) | 0.052 |
| ^†^Systemic vascular resistance (dynes*s/cm^5^) | 1.03 (0.63,1.70) | 0.90 |
| ^†^Pulmonary vascular resistance (dynes*s/cm^5^) | 0.57 (0.08,3.98) | 0.58 |
| **Laboratory Assessment at Shock Onset** |  |  |
| Hemoglobin (g/dL) | 1.07 (0.99,1.16) | 0.09 |
| Serum creatinine (mg/dL) | 0.71 (0.56,0.88) | 0.003 |
| ^†^Aspartate transaminase (mg/dL) | 0.67 (0.40,1.10) | 0.11 |
| ^†^Alanine transaminase (mg/dL) | 0.63 (0.36,1.10) | 0.11 |
| Total bilirubin (mg/dL) | 1.02 (0.76,1.39) | 0.88 |
| ^†^Blood glucose (mg/dL) | 0.18 (0.03,1.05) | 0.06 |
| ^†^B-type natriuretic peptide (pg/mL) | 0.94 (0.85,1.04) | 0.24 |
| Log(Lactate) (mg/dL) | 0.58 (0.43,0.78) | <0.001 |
| Troponin I (ng/mL) | 1.00 (1.00,1.01) | 0.39 |
| ^†^Lactate dehydrogenase (IU/L) | 0.94 (0.73,1.21) | 0.63 |
| pH | 4.12 (0.80,21.12) | 0.09 |
| Bicarbonate (mmol/L) | 1.04 (0.99,1.09) | 0.15 |
| ^†^Arterial partial pressure of oxygen (mmHg) | 3.11 (0.13,77.12) | 0.49 |
| Fraction of inspired oxygen (%) | 0.98 (0.98,0.99) | <0.001 |
| **Echocardiographic Assessment at Shock Onset** |  |  |
| Left ventricle ejection fraction (%) | 1.01 (0.99,1.03) | 0.39 |
| Left ventricle end-diastolic diameter (cm) | 1.07 (0.77,1.49) | 0.69 |
| Mitral valve regurgitation (none/trivial/mild vs. moderate/severe) | 1.52 (0.72,3.24) | 0.28 |
| Tricuspid regurgitation (none/trivial/mild vs. moderate/severe) | 0.88 (0.40,1.95) | 0.75 |
| Right ventricle systolic function (moderately/severely decreased vs. hyperdynamic/normal/low normal/mildly decreased) | 0.79 (0.46,1.37) | 0.40 |
| Tricuspid annular plane systolic excursion (mm) | 1.05 (0.96,1.13) | 0.30 |
| **Left Heart Catheterization and Revascularization Data** |  |  |
| LHC (yes vs. no) | 1.45 (0.56,3.77) | 0.45 |
| ^**^LHC location hospital (University of Utah vs. outside) | 0.82 (0.50,1.33) | 0.42 |
| ^**^Coronary artery dominance (left vs. codominant) | 2.07 (0.67,6.38) | 0.20 |
| ^**^Coronary artery dominance (right vs. codominant) | 1.69 (0.77,3.75) | 0.19 |
| ^**^Left main artery disease (yes vs. no) | 0.56 (0.31,1.03) | 0.06 |
| ^**^Left anterior descending artery disease (yes vs. no) | 0.93 (0.49,1.73) | 0.81 |
| ^**^Left circumflex artery disease (yes vs. no) | 1.02 (0.64,1.61) | 0.94 |
| ^**^Right coronary artery disease (yes vs. no) | 0.82 (0.51,1.32) | 0.41 |
| ^**^Multivessel disease | 0.70 (0.44,1.12) | 0.14 |
| ^**^Diseased vessels number | 0.87 (0.70,1.09) | 0.22 |
| ^**^Culprit vessel (right coronary vs. left anterior descending) | 0.86 (0.46,1.64) | 0.65 |
| ^**^Culprit vessel (left circumflex vs. left anterior descending) | 0.75 (0.35,1.60) | 0.45 |
| ^**^Culprit vessel (other artery vs. left anterior descending) | 0.41 (0.08,2.24) | 0.31 |
| ^**^Culprit vessel (unidentified artery vs. left anterior descending) | 0.55 (0.31,0.98) | 0.044 |
| Coronary intervention (yes vs. no) | 2.94 (1.50,5.76) | 0.002 |
| ^**^Number of stents | 0.77 (0.60,0.98) | 0.034 |
| ^**^Referral for coronary artery bypass graft surgery (yes vs. no) | 1.48 (0.70,3.16) | 0.31 |
| **Complications** |  |  |
| Bleeding complications (yes vs. no) | 0.45 (0.28,0.73) | 0.001 |
| Type 3 bleeding (yes vs. no) | 0.45 (0.28,0.73) | 0.001 |
| Type 4 bleeding (only MCS patients) (yes vs. no) | 0.50 (0.27,0.94) | 0.031 |
| Vascular Complications (yes vs. no) | 0.41 (0.18,0.94) | 0.036 |
| Vascular repair (yes vs. no) | 0.44 (0.18,1.08) | 0.07 |
| Fasciotomy (yes vs. no) | 0.65 (0.22,1.91) | 0.43 |
| Neurologic Complications (yes vs. no) | 1.06 (0.52,2.19) | 0.87 |
| Ischemic stroke (yes vs. no) | 1.08 (0.51,2.27) | 0.84 |
| Hemolysis (only MCS patients) (yes vs. no) | 1.62 (0.87,3.00) | 0.13 |
| Acute kidney injury requiring renal replacement therapy (yes vs. no) | 0.61 (0.35,1.07) | 0.09 |

^*^Variables evaluated together. ^**^These variables only apply to a subset of patients (meeting certain previous conditions as in Table 1). Odds ratios presented are per unit increase for continuous variables (except variables indicated with ^†^; per 1000-unit increase)

**Figure S1.** All-cause Mortality Up To 1-year Following Cardiogenic Shock Presentation Stratified by SCAI Stage

**
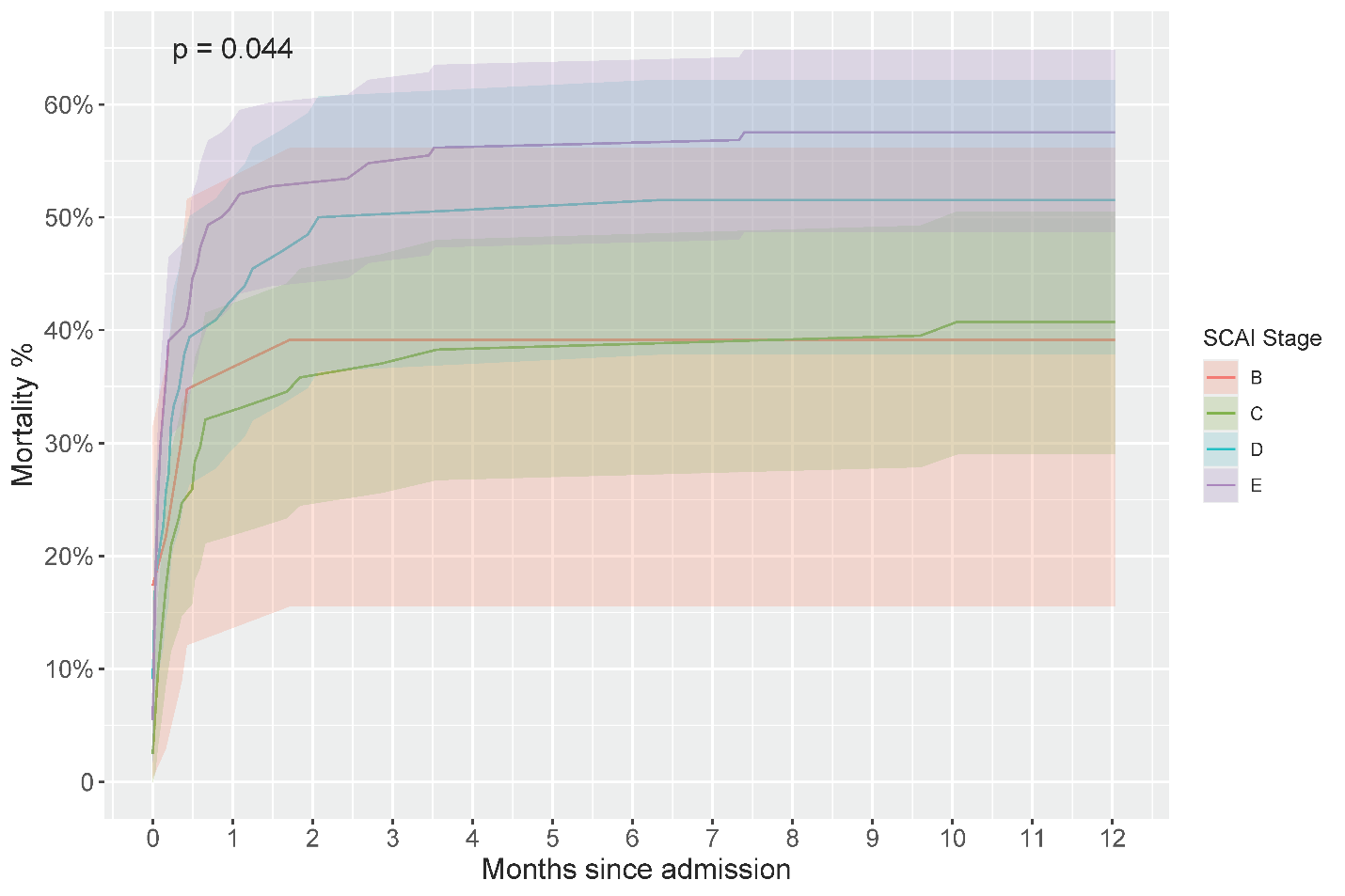
**

**Figure S2.** All-cause Mortality Up To 1-year Following Cardiogenic Shock Presentation Stratified by Myocardial Infarction Location (Left anterior descending artery vs. Other coronary artery)

**
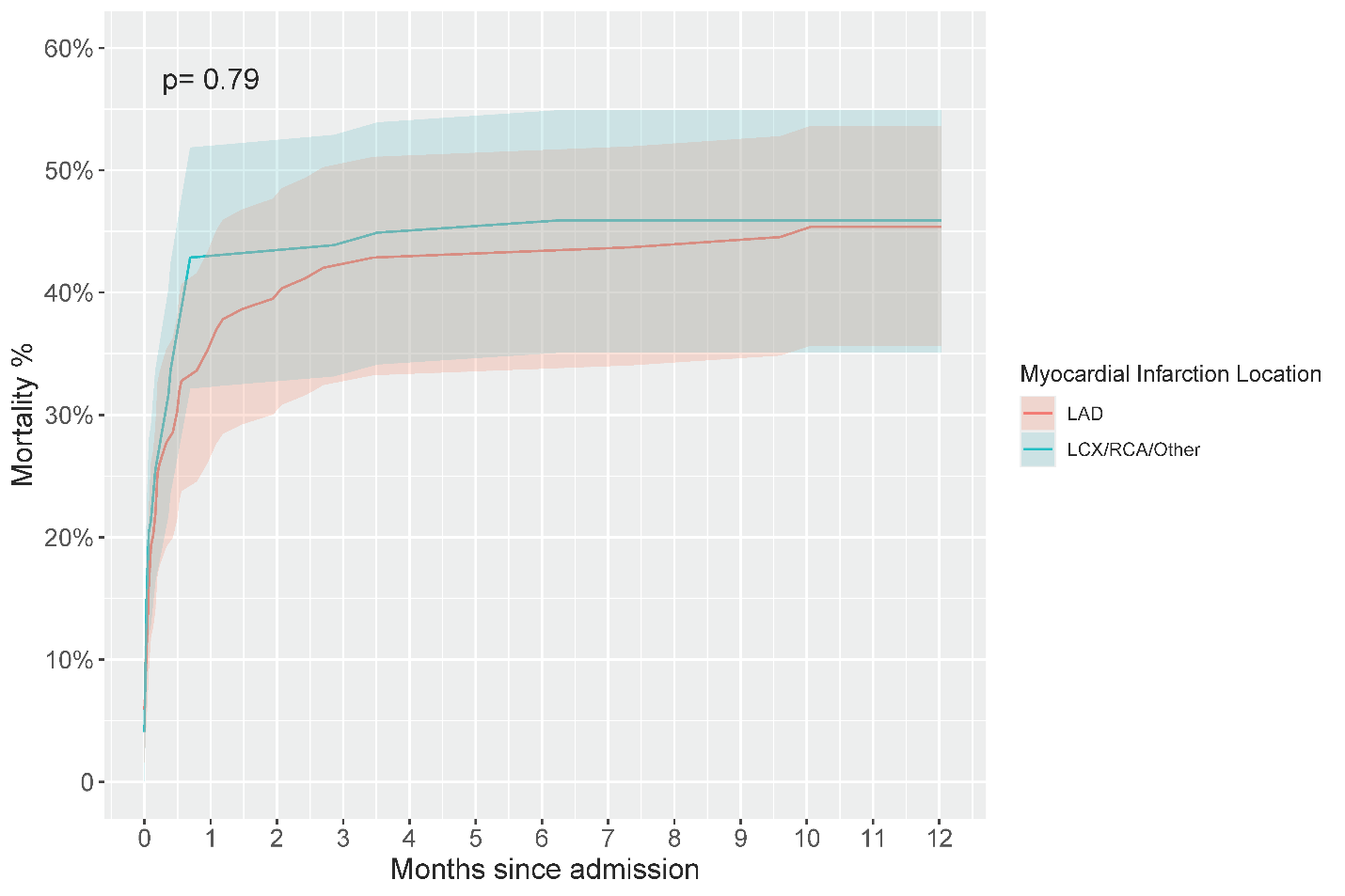
**
